# Gender-specific changes in energy metabolism and protein degradation as major pathways affected in livers of mice treated with ibuprofen

**DOI:** 10.1038/s41598-020-60053-y

**Published:** 2020-02-25

**Authors:** Shuchita Tiwari, Manish Mishra, Michelle R. Salemi, Brett S. Phinney, Joanne L. Newens, Aldrin V. Gomes

**Affiliations:** 1 Department of Neurobiology, Physiology, and Behavior, University of California, Davis, CA USA; 2 Proteomics Core Facility, University of California, Davis, CA USA; 3 Department of Physiology and Membrane Biology, University of California, Davis, CA USA

**Keywords:** Proteomics, Metabolism

## Abstract

Ibuprofen, an inhibitor of prostanoid biosynthesis, is a common pharmacological agent used for the management of pain, inflammation and fever. However, the chronic use of ibuprofen at high doses is associated with increased risk for cardiovascular, renal, gastrointestinal and liver injuries. The underlying mechanisms of ibuprofen-mediated effects on liver remain unclear. To determine the mechanisms and signaling pathways affected by ibuprofen (100 mg/kg/day for seven days), we performed proteomic profiling of male mice liver with quantitative liquid chromatography tandem mass spectrometry (LC-MS/MS) using ten-plex tandem mass tag (TMT) labeling. More than 300 proteins were significantly altered between the control and ibuprofen-treated groups. The data suggests that several major pathways including (1) energy metabolism, (2) protein degradation, (3) fatty acid metabolism and (4) antioxidant system are altered in livers from ibuprofen treated mice. Independent validation of protein changes in energy metabolism and the antioxidant system was carried out by Western blotting and showed sex-related differences. Proteasome and immunoproteasome activity/expression assays showed ibuprofen induced gender-specific proteasome and immunoproteasome dysfunction in liver. The study observed multifactorial gender-specific ibuprofen-mediated effects on mice liver and suggests that males and females are affected differently by ibuprofen.

## Introduction

Ibuprofen, a propionic acid derivative, is the most common over-the-counter nonsteroidal anti-inflammatory (NSAID) drug used to treat fever, pain, inflammation and many other disorders^1^. It has been included as a major medicine in the *Essential Drugs List* of the World Health Organization^2^. Ibuprofen at low over-the-counter doses (800–1200 mg/day) is used to treat muscular aches, backaches, toothaches, and fever. At higher doses (1800–2400 mg/day), ibuprofen is prescribed for the treatment of chronic conditions such as osteoarthritis, rheumatoid arthritis, and ankylosing spondylitis^3^. Ibuprofen is a non-selective inhibitor of the cyclo-oxygenase (COX) isozymes COX-1 and COX-2 that converts arachidonic acid into prostaglandins including thromboxane and prostacyclin^1^. The anti-inflammatory, antipyretic and analgesic effects of ibuprofen are mediated through the inhibition of prostaglandins E2 (PGE2) and I2 (PGI2) production by blocking COX activity^1^. Both PGE2 and PGI2 are pro-inflammatory prostanoids that increase vascular permeability, promote leukocyte infiltration and increase edema formation^4^. The clearance of ibuprofen is mediated through oxidative metabolism by multiple cytochrome P450 (CYP) enzymes (CYP2C9, CYP2C8) to inactive primary metabolites including 3-hydroxy-ibuprofen, carboxy ibuprofen and 2-hydroxy-ibuprofen^5,6^. Most of the ibuprofen is metabolized in liver, while only a small percentage of unchanged drug is excreted in the urine^5^.

The liver plays a key role in energy metabolism and is essential for whole body homeostasis via the regulation of glucose, lipid, and amino acid metabolism^7^. While various NSAIDs, including ibuprofen are considered safe to sell without prescriptions, they have the potential to cause adverse side effects. Adverse reactions associated with NSAIDs treatment can affect the gastrointestinal system, increase blood pressure and cause cardiac, renal and hepatic injuries^8,9^. Treatment with aspirin to a patient with pericarditis was reported to develop acute liver injury^10^. The long term administration (4 weeks) of aspirin and ibuprofen to rats increased mitochondrial number, altered liver mitochondria ultrastructure and increased the metabolic activity of the CYP450 enzymes^11^. Previous research in our laboratory has suggested that physiological concentrations of NSAIDs (diclofenac, naproxen and meclofenamate sodium) cause mitochondrial and proteasome dysfunction in cardiac tissue^12,13^.

To investigate the mechanisms by which ibuprofen affect mouse liver, a quantitative LC-MS/MS technique using tandem mass tag (TMT) labeling was carried out to measure whole liver proteome changes after short-term (7 days) treatment of ibuprofen (100 mg/kg/day). Although the mass spectrometry was initially carried out on livers from male mice, we hypothesized that ibuprofen would cause similar effects on livers from female mice. To determine if ibuprofen has the same effect in male and female mice, biological assays and Western blots were carried out on ibuprofen and vehicle treated mice. The amount of ibuprofen utilized in mice would be equivalent to a human taking approximately 486 mg/day, significantly less than the 1200 mg/day maximum over-the-counter dosage recommended. Ibuprofen at a moderate amount (100 mg/kg/day) affected more proteins and pathways in mice liver than expected, including glycolysis, protein degradation, fatty acid metabolism and antioxidant system. Biological assays and Western blotting exposed significant gender-specific differences in livers from ibuprofen treated males and females relative to their respective controls.

## Materials and Methods

### Animal studies

Aged matched male and female C57BL/6J mice were used for the study. The study protocol was approved by Institutional Animal Care and Use Committee (IACUC) of the University of California, Davis. Mice were maintained at controlled temperature and humidity and had free access to food and water. Methods used for all mice were performed in accordance with UC Davis IACUC guidelines and regulations. Ibuprofen was administered orally in drinking water at a concentration 100 mg/kg/day for seven days. The average water consumption by mice was measured to ensure that mice got the appropriate levels of ibuprofen. The ibuprofen dose used in this study was similar to the concentrations used in previous studies^1,14,15^. This dosage was chosen as it approximates to a human taking 486 mg/day which is between two to three dosages of 200 mg/day. In humans the recommended dosages of ibuprofen are 200 to 400 mg orally every 4 to 6 hours as needed with a maximum dose of 1200 mg/day for over-the-counter use. A 1200 mg/day ibuprofen dose (20 mg/kg/day, assuming an average human weight of 60 kg), would correspond to a treatment of 246.9 mg/kg/day in mice using the dose translation equation (Human equivalent dose (HED) = Animal Dose (mg/kg) × Animal Km (3 for mouse)/Human Km (37 for human)^16^. The maximum human prescription dose (3200 mg/day) would correspond to a 658.4 mg/kg/day dose in mice. Hence, the amount of ibuprofen used in this study (100 mg/kg/day) would approximate to a moderate daily dose of ibuprofen in humans. At the end of the study the mice were euthanized, and the livers were collected and quickly washed twice in ice-cold phosphate-buffered saline (PBS). The tissues were then frozen and pulverized in liquid nitrogen. The pulverized tissue was collected in clean microcentrifuge tubes and stored at −80 °C until needed.

### LC-MS/MS using 10–plex TMT proteomics

#### Sample preparation

Pulverized liver tissue (20 mg aliquot) were homogenized in lysis buffer (8 M urea, 50 mM TEAB (triethyl ammonium bicarbonate), pH 8) followed by sonication (at level 5; three cycles for 10 sec with 30 sec wait in between on ice) to reduce sample viscosity. The lysate was centrifuged at 15,000 g for 15 minutes at 4 °C, and the supernatant was separated. The protein concentration of the supernatant was measured using Bicinchoninic Acid (BCA) protein assay. 100 µg (100 µl) of protein sample was removed and 5 µl of the 200 mM TCEP (tris(2-carboxyethyl) phosphine) added and incubated at 55 °C for 1 hr in a heat block. After that, 5 µl of 375 mM iodoacetamide was added to the sample and incubated for 30 minutes sheltered from light at room temp. The samples were acetone precipitated using pre-chilled (−20 °C) 6 volumes (~600 µL) of acetone overnight. Next day samples were centrifuged at 10,000 xg for 10 minutes at 4 °C, the supernatant was discarded, and pellet was air dried.

#### Digestion of protein samples

The acetone-precipitated protein pellet was resuspended in 100 µl of 50 mM ammonium bicarbonate (NH_4_CO_3_). 2.5 µL of trypsin (i.e. 2.5 µg) per 100 µg of protein was added to each sample and were incubated overnight at 37 °C. The digested samples were lyophilized with a speedvac and resuspended in 0.1% trifluoroacetic acid (TFA) and 5% acetonitrile.

#### TMT 10-plex peptide labeling

TMT label reagents were allowed to equilibrate to room temperature (RT) and anhydrous acetonitrile was added to each tube. 21.6 µL of the TMT label reagents were added to each 20 µg of samples and were incubated for 1 hr at RT. The reaction was quenched by adding 8 µL of 5% hydroxylamine to the labeled samples and were incubated for 30 minutes at RT. All TMT labeled samples were combined in equal amounts in a new tube (200 µg) and were run on Mass spec after fractionation.

TMT labeled samples were then reconstituted in 0.1% TFA and the pH adjusted to 2 with 10% TFA^17^. The combined sample (200 µg) was separated into 8 fractions by Pierce High pH Reverse-Phase Peptide Fractionation kit (Thermo Scientific) with an extra wash before separation to remove extra labels^17^. The eight fractions were dried almost to completion.

#### LC-MS/MS

LC separation was done on a Dionex Nano Ultimate 3000 (Thermo Scientific) with a Thermo Easy-Spray source^17^. The digested peptides were reconstituted in 2% acetonitrile/0.1% TFA and 5 µl of each sample was loaded onto a PepMap 100 Å 3U 75 µm × 20 mm reverse-phase trap where they were desalted online before being separated on a 100 Å 2U 50-micron × 150 mm PepMap EasySpray reverse-phase column^17^. Peptides were eluted using a 180-minute gradient of 0.1% formic acid (A) and 80% acetonitrile (B) with a flow rate of 200 nL/min. The separation gradient was run with 2% to 5% B over 1 minute, 5% to 10% B over 9 minutes, 10% to 20% B over for 27 minutes, 20% to 35% B over 10 minutes, 35% B to 99% B over 10 minutes, a 2 minute hold at 99% B, and finally 99% B to 2% B held at 2% B for 5 minutes^17^.

#### MS3 synchronous precursor selection workflow

Mass spectra were collected on a Fusion Lumos mass spectrometer (Thermo Fisher Scientific) in a data-dependent MS3 synchronous precursor selection (SPS) method^17^. MS1 spectra were acquired in the Orbitrap, 120 K resolution, 50 ms max inject time, 5 ×10^5^ max inject time. MS2 spectra were acquired in the linear ion trap with a 0.7 Da isolation window, CID fragmentation energy of 35%, turbo scan speed, 50 ms max inject time, 1 ×10^4^ automatic gain control (AGC) and maximum parallelizable time turned on^17^. MS2 ions were isolated in the ion trap and fragmented with a HCD energy of 65%. MS3 spectra were acquired in the orbitrap with a resolution of 50K and a scan range of 100–500 Da, 105 ms max inject time and 1 × 10^5^ AGC^17^.

#### TMT data analysis (Quantitative data analysis)

The use of 5 ibuprofen treated samples and 5 vehicle treated samples for the TMT 10 plex experiment animals and a [1.2]-fold cut-off was based on power analysis taking into account that isobaric-labeled LC-MS/MS quantitative measurements are known to be compressed by various factors. These factors include ratio compression and cause an underestimation of changes in relative abundance of proteins across samples^18^. This underestimation was ≥50% for other isobaric labeling-based experiments^19^. In these experiments a protein that was upregulated by 2.4 gave a measurement of only 1.2^19^. As such the [1.2]-fold change for the TMT experiment was considered to be equivalent to a [1.5]-fold change. For power analysis (using online program https://www.stat.ubc.ca/rollin/stats/ssize/n2.html), when sigma (common standard deviation) was set at 25%, power at 0.80 and α = 0.05, a minimum of 4 mice in each group was needed.

Raw files were processed with Proteome Discoverer 2.2 (Thermo Scientific) using the default MS3 SPS method with the following modifications for importation into Scaffold Q+ . Target Decoy PSM validator was used instead of Percolator and Maximum Delta Cn was set to 1. All MS/MS samples were analyzed using Sequest HT to search all mouse sequences from Uniprot (https://www.uniprot.org/proteomes/UP000000589) and 110 common laboratory contaminants (http://thegpm.org.crap) plus an equal number of reverse decoy sequences assuming the digestion enzyme trypsin^17^. Sequest-HT was searched with a fragment ion mass tolerance of 0.20 Da and a parent ion tolerance of 10.0 PPM. Carbamidomethyl of cysteine and TMT10 plex of lysine were specified in Sequest-HT as fixed modifications^17^. Oxidation of methionine and acetyl of the n-terminus were specified in Sequest-HT as variable modifications.

Scaffold Q+ (version Scaffold_4.9.0, Proteome Software Inc., Portland, OR) was used to quantitate Label Based Quantitation (iTRAQ, TMT, SILAC, etc.) peptide and protein identifications. Peptide identifications were accepted^17^ with a decoy false discovery rate (FDR) cutoff of less than 0.2%. Protein identifications were accepted if they could be established with at least 2 unique identified peptides. Proteins that contained similar peptides and could not be differentiated based on MS/MS analysis alone were grouped to satisfy the principles of parsimony. Proteins sharing significant peptide evidence were grouped into clusters^20^. Channels were corrected as described in the supplementary text^21^. Normalization was performed iteratively (across samples and spectra) on intensities, as described in Statistical Analysis of Relative Labeled Mass Spectrometry Data from Complex Samples using ANOVA^22^. Medians were used for averaging. Spectra data were log-transformed and weighted by an adaptive intensity weighting algorithm. Of 80801 spectra in the experiment at the required thresholds, 79849 (99%) were incorporated into the quantitation and 4109 proteins were detected in 3700 clusters with 6 decoys (FDR 0.1%). 477 proteins were found to be differentially expressed (Supplemental Table 1), of which 302 proteins had a minimum fold change of 1.2 (Supplemental Table 2). All the analysis was carried out using the information from the 302 differentially expressed proteins (0.3% FDR) which show ≥[1.2]-fold change.

### 26S proteasome activity assay

**Figure 1:**
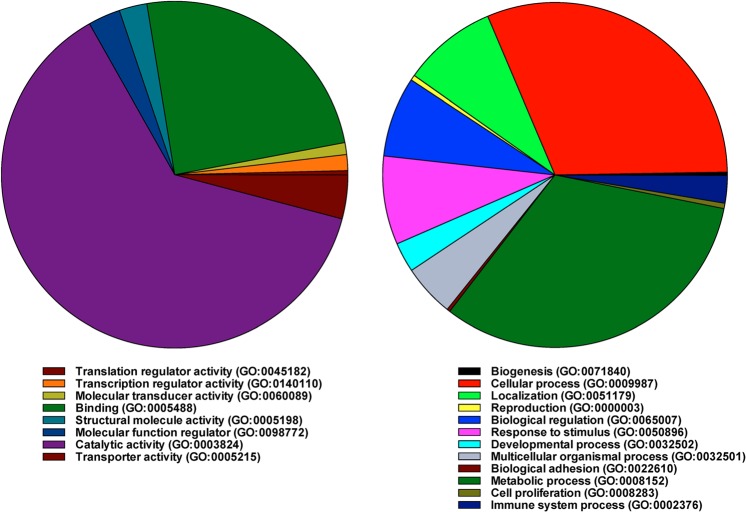
A pie chart depicting the proteomic analysis of control and ibuprofen-treated male mice liver. Biological processes associated with the differentially expressed proteins were shown using the PANTHER classification system.

Briefly, pulverized liver samples (20 mg aliquot) were homogenized in 26 S proteasome lysis buffer (50 mM Tris, 150 mM sodium chloride (NaCl), 1 mM ethylenediaminetetraacetic acid (EDTA), 5 mM magnesium chloride (MgCl_2)_ [pH 7.5]) with a hand-held Potter-Elvehjem homogenizer. The supernatant containing the proteasomes were separated after centrifugation at 12,000 xg for 15 min at 4 °C and were quantified and diluted to 1 μg/μL concentration with proteasome lysis buffer. The β5 (chymotrypsin-like), β2 (trypsin-like) and β1 (caspase-like) activities were assayed using 20 µg of protein. The assays were carried out in a total volume of 100 µL per well in a black 96-well plate. The adenosine triphosphate (ATP)-dependent 26 S assays were performed using 100 μM ATP in the absence and presence of a specific proteasome inhibitor: 10 μM bortezomib (β5 activity) and 100 μM bortezomib (β1 and β2 activities). Specific fluorescence-tagged substrates for each proteasomal β subunits (Enzo Life Sciences, NY, USA): Z-LLE-AMC for β1, Boc-LSTR-AMC for β2 and Suc-LLVY-AMC for β5 were used to initiate the reaction [27–30]. Proteasomal activities were measured at an excitation wavelength of 390 nm and an emission wavelength of 460 nm using a Fluoroskan Ascent fluorometer (Thermo Electron, MA) for every 15 min up to 120 min. Controls to validate the inhibitors used for these experiments are shown in Supplemetary Fig. 1, and more extensive details about the proteasome assays have been previously published^23^.

### Immunoproteasome activity

20 μg of liver samples from ibuprofen-treated and control animals were incubated with immunoproteasome buffer (50 mM Tris, 5 mM MgCl_2_, 20 mM potassium chloride (KCl), 1 mM DTT (freshly added), pH 7.5), and inhibitor or an equal volume of dimethyl sulfoxide (DMSO) for 20 min at room temperature. The specific inhibitors for β5i 20 µM ONX-0914 (Abmole Bioscience Inc., Houston, TX, Cat. No. M2071) and for β1i 50 µM bortezomib was used to evaluate the specificity of the assay. The 25 µM fluorogenic substrates ANW-R110 (AAT Bioquest, Inc, CA) for β5i and (Ac-Ala-Asn-Trp)2-R110 (AAT Bioquest, Cat. No. 13455) for β1i were used to initiate the reaction. The fluorescence intensity was measured every 5 mins for 60 min at an excitation of 498 nm and an emission of 520 nm at 37 °C in a Tecan Infinite M1000.

### Catalase activity assay

10 µg of mice liver was homogenized in 1.15% KCl using a Dounce homogenizer. The samples were centrifuged at 12,000 rpm for 15 minutes. The supernatant was extracted and diluted with 50 mM phosphate buffer pH 7.0. The colorimetric assay to measure catalase activity in liver samples was done by using Sigma-Aldrich Catalase Assay Kit (Cat. CAT100). This assay in the presence of hydrogen peroxide (H_2_O_2_) and horseradish peroxidase (HRP) uses a substituted phenol (3,5-dichloro-2-hydroxybenzenesulfonic acid) that combines with 4-aminoantipyrine to form a red quinoneimine dye. All solutions were prepared as instructed by the manufacturer. Briefly, this assay measured the H_2_O_2_ substrate that remains after the catalase action. In first step, catalase via its catalytic pathway converted H_2_O_2_ to water and oxygen, and the reaction was stopped using sodium azide. Next, a small volume of reaction mix is then analyzed for the amount of H_2_O_2_ remaining by a colorimetric method. The absorbance was measured using a spectrophotometer at 520 nm, and the actual concentration was measured using Beer’s Law: [H_2_O_2_] (mM) = A240/0.0436.

### Hydrogen peroxide (H_2_O_2_) assay

H_2_O_2_ assay was performed using Sigma Aldrich Fluorimetric Hydrogen Peroxide Assay Kit, (Cat. # MAK165). All steps were carried out as per manufacturer’s instructions. The assay is based on the peroxidase substrate that reacts with H_2_O_2_ to generate a red fluorescent product that can be analyzed by a fluorescent microplate reader. Briefly, controls, standards, and samples were added to the wells followed by the addition of a master mix that contains red peroxidase substrate, peroxidase and assay buffer. The plate was incubated for 15 minutes at room temperature protected from light. The fluorescence intensity was measured at excitation = 540 and emission = 590 nm.

### Western blotting

#### Sample preparation

Pulverized liver tissue (20 mg) was homogenized in ice cold 1x RIPA buffer (50 mM Tris, 150 mM NaCl, 1% NP40, 0.5% sodium deoxycholate and 0.1% sodium dodecyl sulfate (SDS), pH 8) using a glass dounce homogenizer. The homogenates were centrifuged at 12,000 xg for 15 min at 4 °C. The supernatants were separated and quantified using the BCA protein assay (Bio-Rad, Cat. #500–0119) and were diluted to equal protein concentrations. Liver samples were then mixed with 4X Laemmli sample buffer (8% SDS, 40% glycerol, 0.4% bromophenol blue, 240 mM Tris, pH 6.8) with freshly added β-mercaptoethanol and were heated for 5 mins at 95 °C.

#### Electrophoresis and western blotting

Equal amounts of protein (20 µg) were separated on 4–20% 18-well TGX precast gels (Cat. # 567–1094, Bio-Rad) and were transferred to a nitrocellulose membrane (Trans-Blot Turbo Midi Nitrocellulose, #170–4159, Bio-Rad) using the Trans-Blot Turbo Transfer System (Cat # 170–4155, Bio-Rad). The membranes were then stained with Ponceau S and imaged to serve as a loading control for total protein normalization of Western blots. Thereafter the membrane was blocked with 3% nonfat dry milk (NFM) (Cat. # 170–6404, Bio-Rad) in Tris-buffered saline (TBS) (50 mM Tris, 150 mM NaCl, pH 7.5) containing 0.05% (wt/vol) Tween 20 (TBST) at room temperature. The membranes were then incubated overnight at 4 °C with the following 14 primary antibodies diluted to 1:2000 in 1% TBST: mouse anti-CYP1A2 (sc-53614), mouse anti-PFK-1 (sc-166722), mouse anti-fatty acid synthase (A-5) (sc-55580), mouse anti-PKLR (sc-133222), mouse anti-GSTM1 (sc-517262), mouse anti-CROT (H-1)(sc-365976), mouse anti-PROHIBITIN 2 (A-2) (sc-133094), mouse anti-ATP5F1 (C-12) (sc-514419), mouse anti-CYP4A1(clo1) (sc-53248), mouse anti-PKM (C-11) (sc-365684), mouse anti-GSTT2 (D-1) (sc-514667), mouse anti-FAAH (27-γ) (sc-100739), mouse anti-17-βHSD2 (E-7) (sc-374150), mouse anti-GSTA1/2/5 (E-6) (sc-398714), mouse anti-ETFDH (D-2) (sc-515202), mouse anti-LFABP (C-4) (sc-374537), mouse anti-LMP2,β1i (A-1) (sc-271354, LOT#B1611), mouse anti-MECL-1(71P6) β2i (sc-130295) (all from Santa Cruz), mouse anti-β5i (1:2000, custom made by Abgent), and rabbit anti-PSMA6 (1:20,000; Epitomics), The membranes were washed three times in TBST for 5 mins each and were incubated at room temperature for 1 hr with horseradish peroxidase conjugated rabbit anti-mouse or anti-rabbit IgG secondary antibody (Sigma-Aldrich, anti-mouse Cat. # A9044, anti-rabbit Cat. # A0545) diluted 1:10,000 in 1% TBST. Blots were subsequently washed three times with 1X TBST for 5 mins each and developed using a commercial chemiluminescent reagent (Clarity, Bio-Rad-170–5061) and imaged using the ChemiDoc MP (Bio-Rad). All incubation steps carried out at 4 °C or RT were done with gentle shaking. The quantification of blots was done by using Image Lab 5.0.

### Statistical analysis

A summary of the number of samples used for the different experiments in these studies is shown in Supplemental Table 3.

#### For proteomics

Differentially expressed proteins were determined by applying Permutation Test with unadjusted significance level of p < 0.05 corrected by the Benjamini-Hochberg procedure to reduce the FDR (p < 0.00674 after using Benjamini-Hochberg procedure).

#### For western blotting and biological assays

Results are expressed as mean ± standard deviation (SD) from at least three independent experiments. The comparisons between groups were performed using the Student’s t-test or one-way ANOVA. P values of <0.05 were defined as statistically significant.

## Results

Proteomic profiling of male mice liver with quantitative 10-plex TMT LC-MS/MS showed significant differences between 7-day vehicle and ibuprofen treated mice livers. To our knowledge, this is the first report of proteomic profiling of mice liver treated with ibuprofen.

### Metabolic processes

The quantitative data obtained from mass spectrometry were analyzed using Panther program (http://www.pantherdb.org/about.jsp). The data revealed that when differentially expressed proteins were grouped by biological process, proteins associated with metabolic processes (GO:0008152) were the largest group of proteins that were altered (32.4%) in male ibuprofen-treated mice (Fig. 1). The other major biological process with the most proteins altered was cellular processes (GO:0009987) (Fig. 1). Based on molecular function, the comparison between the control and the ibuprofen treated mice livers showed that around 62.7% of the altered proteins were associated with catalytic activity (GO:0003824) (Fig. 1). Using the Panther Go-Slim program option, twenty-three biological processes showed a P-value of <0.001 and an FDR of <0.5% for over or underrepresented differentially expressed proteins (Table 1). These processes included fatty acid, carbohydrate, steroid, coenzyme, acyl-CoA metabolic processes, respiratory electron transport chain, G protein-coupled receptor pathway, and cell surface receptor signaling.

**Table 1:** Biological processes in mice liver affected by ibuprofen treatment.

PANTHER GO-Slim Biological Process	Number of differentially expressed proteins	Expected	Fold Enrichment	+/−	Raw P value	False discover rate (FDR)
fatty acid beta-oxidation	7	0.25	27.56	+	2.93E-08	7.94E-07
fatty acid metabolic process	26	2.23	11.67	+	4.67E-19	3.80E-17
lipid metabolic process	44	5.21	8.45	+	1.94E-26	4.75E-24
primary metabolic process	98	55.86	1.75	+	3.18E-09	1.11E-07
metabolic process	125	69.37	1.8	+	2.29E-13	1.40E-11
pentose-phosphate shunt	2	0.08	24.75	+	4.43E-03	3.28E-02
carbohydrate metabolic process	12	3.6	3.33	+	3.73E-04	4.33E-03
vitamin biosynthetic process	3	0.13	23.62	+	4.87E-04	4.95E-03
vitamin metabolic process	3	0.25	11.81	+	2.81E-03	2.14E-02
cellular amino acid biosynthetic process	11	0.72	15.37	+	6.58E-10	2.68E-08
cellular amino acid metabolic process	31	2.62	11.83	+	1.07E-22	1.31E-20
porphyrin-containing compound metabolic process	3	0.24	12.37	+	2.49E-03	1.96E-02
cellular amino acid catabolic process	8	0.66	12.16	+	7.43E-07	1.81E-05
acyl-CoA metabolic process	5	0.43	11.71	+	1.12E-04	1.61E-03
coenzyme metabolic process	12	1.25	9.62	+	1.34E-08	4.08E-07
cholesterol metabolic process	4	0.42	9.62	+	1.10E-03	1.03E-02
steroid metabolic process	14	1.42	9.86	+	6.07E-10	2.96E-08
lipid transport	6	0.84	7.12	+	2.89E-04	3.71E-03
respiratory electron transport chain	8	1.29	6.19	+	7.21E-05	1.17E-03
generation of precursor metabolites and energy	10	2.11	4.73	+	7.90E-05	1.20E-03
sulfur compound metabolic process	5	0.88	5.7	+	2.36E-03	1.98E-02
muscle contraction	7	1.26	5.56	+	3.77E-04	4.18E-03
phospholipid metabolic process	7	2.05	3.41	+	5.48E-03	3.82E-02
catabolic process	33	13.9	2.37	+	5.62E-06	1.25E-04
regulation of biological process	16	38.33	0.42	−	2.77E-05	5.20E-04
biological regulation	25	45.01	0.56	−	6.55E-04	6.39E-03
RNA metabolic process	6	17.73	0.34	−	1.75E-03	1.53E-02
nucleobase-containing compound metabolic process	17	31.29	0.54	−	5.07E-03	3.64E-02
G-protein coupled receptor signaling pathway	0	10.1	<0.01	−	6.12E-05	1.07E-03
cell surface receptor signaling pathway	4	19.96	0.2	−	2.65E-05	5.40E-04
signal transduction	17	33.43	0.51	−	1.41E-03	1.27E-02
cell communication	21	37.74	0.56	−	2.40E-03	1.95E-02
sensory perception of smell	0	8.07	<0.01	−	4.54E-04	4.82E-03
sensory perception of chemical stimulus	0	8.64	<0.01	−	3.09E-04	3.77E-03

Twenty-three biological processes showed a p value of <0.001 and an FDR of <0.5% for over or underrepresented differentially expressed proteins using the Panther program.

### Proteasome dysfunction

**Figure 2:**
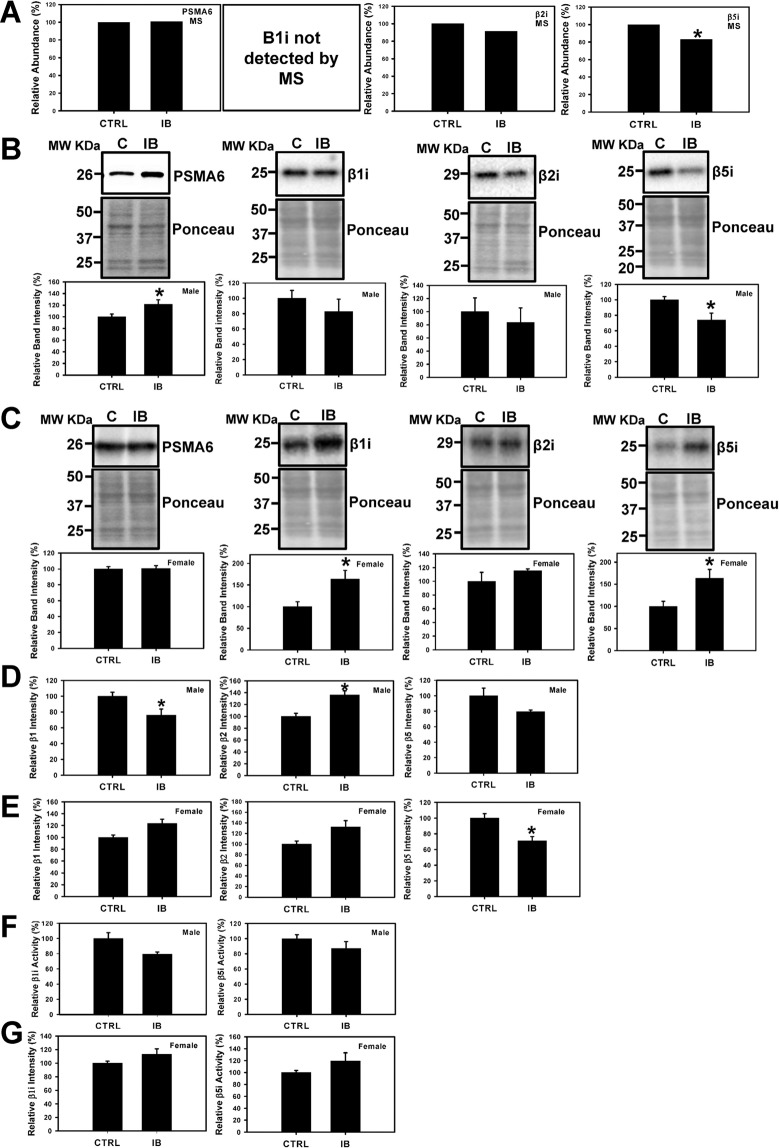
Effects of ibuprofen treatment on 20S proteasome and immuno-proteasome expression levels and activities in mice liver. (**A**) Changes in level of 26S proteasome subunits (β1, β2 and β5) and immunoproteasome subunits (β1i and β5i) obtained by mass spectrometry. Western blots of proteasome levels (PSMA6, β1i, β2i and β5i) and total protein (used as loading controls) in (**B**) Male liver, and (**C**) Female liver. Value are mean ± SE; n = 4–6 per group. *p < 0.05. (**D**) 26S proteasome (β1, β2 and β5) activities of liver lysates from male mice. (**E**) 26S proteasome (β1, β2 and β5) activities of liver lysates from female mice. (**F**,**G**) β1i and β5i immune-proteasome activities of male and female liver lysates. Value are mean ± SE; n = 6 per group. *p < 0.05.

The proteomic data suggested that that the level of the relatively low abundant proteasome subunit beta type-8 (PSMB8) (β5i) was decreased in ibuprofen treated male mice (Supplemental Table 1). During stress conditions, the constitutive proteasome β1, β2 and β5 subunits of 20 S proteasome are replaced by β1i, β2i, and β5i immunoproteasome counterparts^24–26^. Western blotting data showed that the expression levels of β5i immunoproteasome subunit was significantly decreased in ibuprofen treated male livers, consistent with the proteomic data (Fig. 2A,B). However, β5i was significantly increased in ibuprofen treated female livers relative to control (Fig. 2C). The β1i subunit expression was considerably elevated in ibuprofen treated female livers but not in males whereas no change was observed in the levels of β2i expression in both male and female livers (Fig. 2B,C). The expression levels of PSMA6 (20S catalytic core subunit) in the ibuprofen treated male livers was significantly elevated relative to controls whereas no change was observed in female mice livers treated with ibuprofen and controls (Fig. 2B,C). Since changes in proteasome activity are suggested to be associated with various pathological conditions including aging, cardiac and neurodegenerative diseases^27–29^, we investigated the effects of ibuprofen on the proteasome and immunoproteasome activities. The β1 (caspase-like) proteasome activity was significantly decreased in ibuprofen treated male livers while significantly increased in ibuprofen treated female livers compared to control animals (Fig. 2D,E). The β2 (trypsin-like) activity was found to be significantly elevated in male livers, and equally significant (0.050) in ibuprofen-treated female livers relative to normal control livers (Fig. 2D,E). The β5 (chymotrypsin-like) activity was significantly decreased in ibuprofen treated female livers and showed a trend toward decrease (p = 0.085) in ibuprofen treated male livers (Fig. 2D,E). No change in immunoproteasome activities was observed in ibuprofen treated male and female livers compared to normal control livers (Fig. 2F,G).

**Figure 3:**
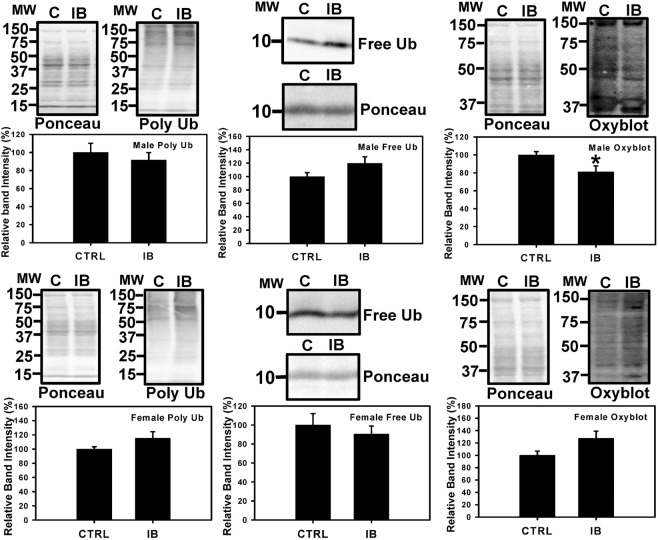
Characterization of levels of polyubiquitination, free ubiquitins and oxidized proteins in male and female mice livers. Western blot of polyubiquinated, free ubiquitin levels and oxidized proteins in total liver lysates from mice (**A**) Male livers & (**B**) Female livers. Value are mean ± SE; n = 6 per group. *p < 0.05.

To further investigate if ibuprofen altered proteasome function enough to change the levels of the ubiquitinated and oxidized proteins, liver lysates were probed with anti-ubiquitin and anti-DNPH antibodies. The levels of polyubiquitinated and free ubiquitin proteins remained unchanged in both ibuprofen treated male and female livers (Fig. 3A,B). However, the levels of oxidized proteins decreased in ibuprofen treated male livers but not in ibuprofen-treated female livers (Fig. 3A).

### Energy production pathways

The proteomic data revealed that several proteins related to fatty acid metabolism and glycolytic pathway as well as other metabolic pathways were either upregulated or downregulated in ibuprofen treated male mice liver compared to control. To better understand the underlying molecular mechanisms, validation of the expression levels of different proteins associated with different energy metabolic pathways was carried out using Western blotting and biochemical assays to determine altered pathways activities.

### Fatty acid metabolism pathway

**Figure 4:**
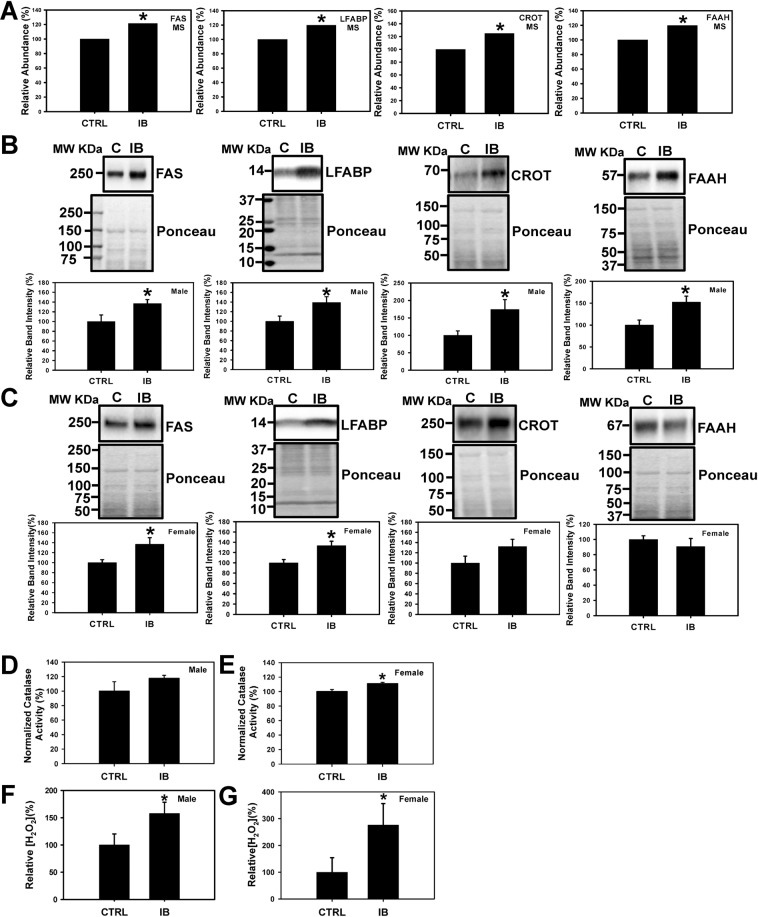
Validation of expression levels of proteins related to fatty acid metabolism pathway in male and female mouse liver. (**A**) **C**hanges in the level of proteins FAS (Fatty Acid Synthase), LFABP (Liver Fatty acid-binding protein), CROT (carnitine O-octanoyltransferase), and FAAH (fatty acid amide hydrolase) obtained from mass spectrometry. Western blots and corresponding semi quantitative bar graphs for proteins, FAS, LFABP, CROT, and FAAH and total protein (used as a loading control) (**B**) Male liver and (**C**) Female liver. Value are mean ± SE; n = 4–6 per group. *p < 0.05. Characterization of antioxidant catalase activity and hydrogen peroxide levels in control and ibuprofen-treated mouse livers. (**D**), (**E**) Catalase activity in male and female mouse liver. (**F**), (**G**) Relative hydrogen peroxide (H_2_O_2_) levels of male and female liver lysates. Value are mean ± SE; n = 5 per group. *p < 0.05.

To validate the expression levels of proteins related to β fatty acid metabolism the total liver lysates from male and female mice were probed with several antibodies to investigate the enzymes involved in fatty acid metabolism. The data suggests that all four proteins FAS (fatty acid synthase), LFABP (liver fatty acid-binding protein), CROT (carnitine O-octanoyltransferase), and FAAH (fatty acid amide hydrolase) (Fig. 4B) were significantly upregulated in male livers, consistent with proteomic data (Fig. 4A), whereas only FAS and LFABP was upregulated in ibuprofen treated female livers (Fig. 4C).

**Figure 5:**
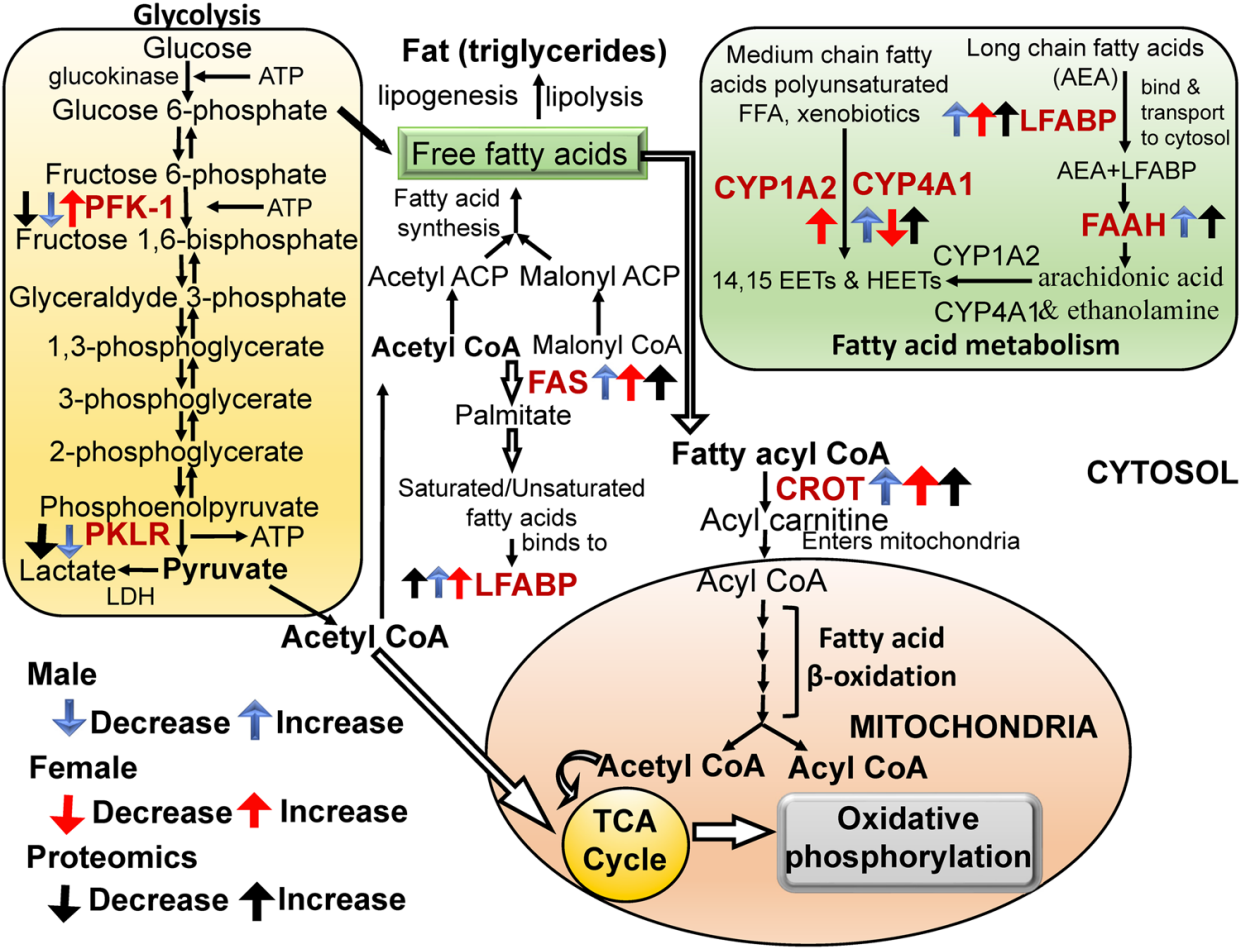
Schematic diagram showing the changes in the levels and expression of enzymes involved in the glycolytic and fatty acid pathways in ibuprofen treated male and female mice livers. Abbreviations: PFK-1 (Phosphofructokinases-1); PKLR (pyruvate kinase, liver and RBC); FAS (Fatty acid synthase); LFABP (Liver Fatty acid-binding protein); FAAH (fatty acid amide hydrolase); CROT(carnitine O-octanoyltransferase); CYP4A1(Cytochrome P450 4A1), ATP(Adenosine triphosphate); CYP1A2 (Cytochrome P450 1A2); TCA (tricarboxylic acid cycle).

Since nearly all the enzymes involved in both mitochondrial β oxidation and peroxisomal β oxidation were upregulated by ibuprofen (Fig. 5, Supplemental Fig. 3), we measured H_2_O_2_ levels in the liver (Fig. 4F,G). H_2_O_2_ are reactive oxygen species that is increased when peroxisomal β oxidation is increased, and functions both as intracellular messenger and as a source of oxidative stress^30^. In mice livers treated with ibuprofen, H_2_O_2_ was found to be significantly increased by 1.56-fold in male and 2.75-fold in females (Fig. 4F,G). Increases in H_2_O_2_ may lead to increases in catalase activity, and we observed significantly increased catalase activity in ibuprofen treated male livers (Fig. 4D) as well as in female mice livers (Fig. 4E).

#### Other enzymes involved in fat and drug metabolism

**Figure 6:**
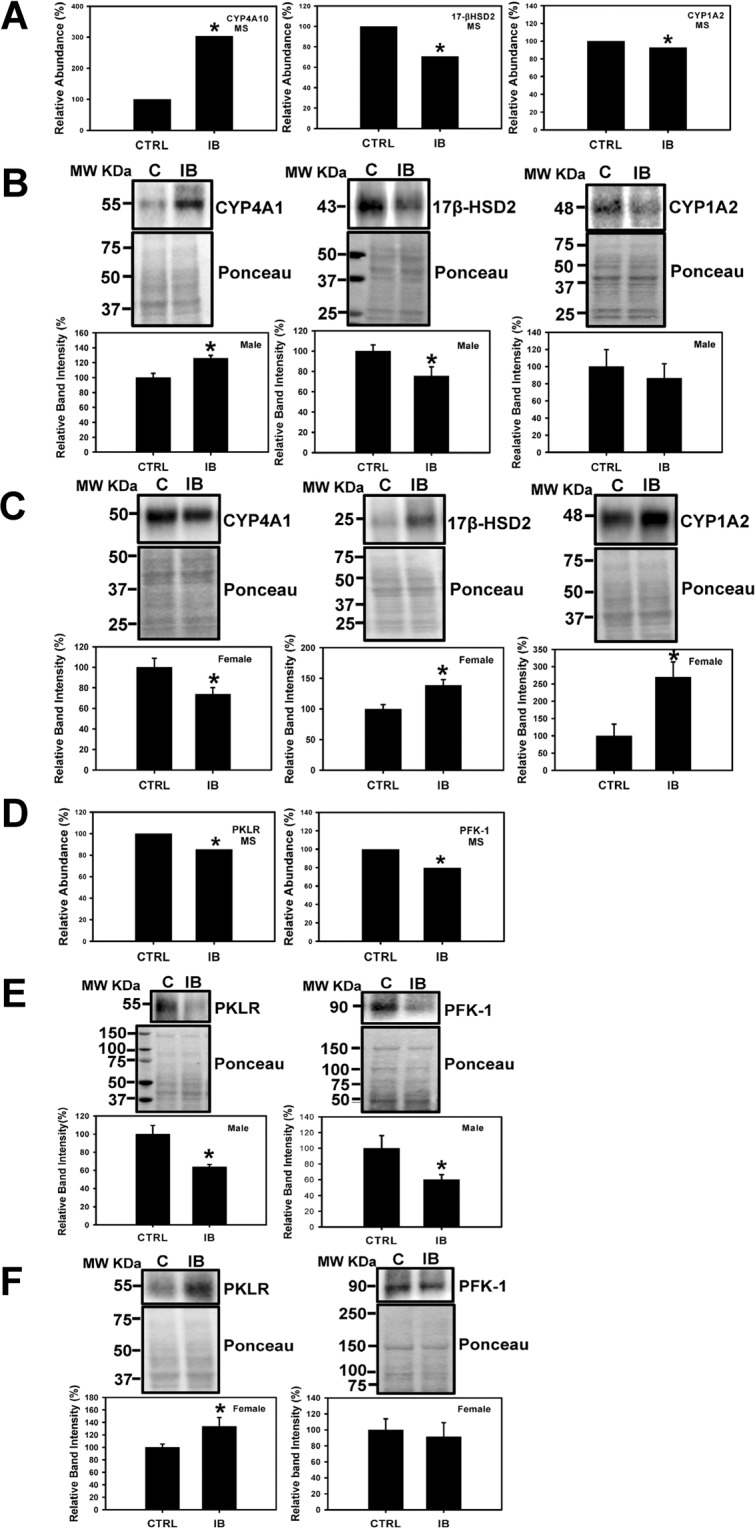
Determination and validation of expression levels of proteins related to fatty acid metabolism and glycolytic pathways in mouse liver. Changes in level of CYP4A10 (Cytochrome P450 4A10), 17β-HSD2 (17β hydroxysteroid dehydrogenase type 2), and CYP1A2 (Cytochrome P450 1A2) obtained by mass spectrometry (**A**). Western blots and corresponding semi quantitative bar graphs for CYP4A1, 17β-HSD2, and CYP1A2 (Cytochrome P450 1A2) and total protein (used as a loading control). (**B**) Male liver and (**C**) Female liver. Value are mean ± SE; n = 4–6 per group. *p < 0.05. (**D**) Proteomic analysis and Western blots of proteins, PKLR (pyruvate kinase, liver and RBC), and PFK-1 (Phosphofructokinases-1), total protein (used as a loading control) and corresponding semi- quantitative bar graphs are shown for (**E**) male mice liver, and (**F**) female mice liver. Value are mean ± SE; n = 4–6 per group. *p < 0.05.

Since proteomic results showed that CYP4A10 (cytochrome P450 A10) and CYP4A14 increased by 1.74 and 3.03-fold in ibuprofen treated male livers and antibodies to these were unavailable, we determined the expression of CYP4A1 which has >90% sequence homology with CYPA10. The expression levels of CYP4A1 was significantly increased, 17β-HSD2 (17β hydroxyl steroid dehydrogenase type 2) was decreased, and CYP4A2 (cytochrome P450 1A2) not statistically changed in ibuprofen treated male livers (Fig. 6B). In contrast, the levels of CYP4A1 was decreased and CYP4A2 and 17β-HSD2 were increased in female livers treated with ibuprofen (Fig. 6C), suggesting a gender-specific difference in mice liver. A summary of some of the data is shown in Fig. 5.

#### Glycolytic pathway

Validation of glycolytic pathway proteins using Western blotting showed that the expression levels of PKLR (pyruvate kinase, liver and RBC), and PFK-1 (Phosphofructokinases-1) was downregulated in male livers than controls (Fig. 6E) while levels of PKLR was upregulated in ibuprofen treated female livers suggesting a gender-specific difference with respect to glycolytic pathway. However, no difference was observed in the levels of PFK-1 in female livers (Fig. 6F).

#### Mitochondrial proteins

Consistent with proteomic data (Supplementary Fig. 2A) expression levels of inner mitochondrial membrane proteins Prohibitin-2 and ETFDH (electron transfer flavoprotein dehydrogenase) were both increased in male livers (Supplementary Fig. 2B). ATP5F1 (mitochondrial ATP synthase 5F1) showed a small increase in the proteomic studies but showed a decrease by Western blotting. The levels of ATP5F1, Prohibitin-2 and ETFDH were significantly increased in female livers treated with ibuprofen (Supplementary Figure 2C).

### Antioxidant defense system

**Figure 7:**
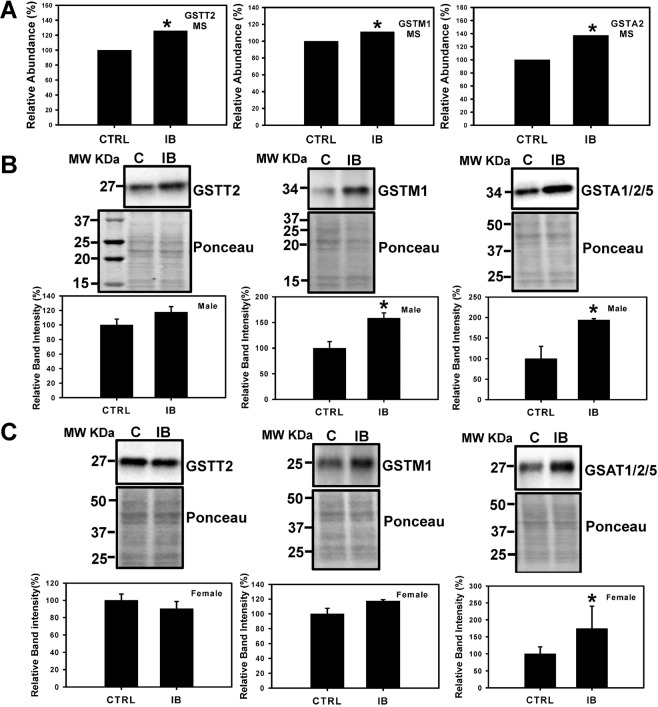
Effect of short-term ibuprofen treatment (7 days) on levels of oxidative-stress related proteins in control and ibuprofen-treated mouse livers. Change in levels of protein associated with oxidative stress, GSTT2 (Glutathione S-transferase theta-2), GSTMI (Glutathione S-transferase mu 1), and GSTA1/2/5 (Glutathione S-transferase alpha 1/2/5) obtained by mass spectrometry (A). Western blot analysis was performed to determine the expression levels of protein associated with oxidative stress, GSTT2, GSTMI, and GSTA1/2/5 in (B) Male mouse livers, and (C) Female mouse livers. Band signal intensities were analyzed by Image Lab® Software Version 12 and normalized with intensities obtained from loading control after staining with commercial Ponceau S for total protein. Value are mean ± SE; n = 4–6 per group. *p < 0.05.

During oxidative stress and other stress conditions increased levels of H_2_O_2_ is associated with elevated production of antioxidant enzymes^31^. The proteomic data showed that various antioxidant enzymes such as glutathione S-transferase theta 2 (GSTT2), glutathione S-transferase Mu 1 (GSTMI), and glutathione S-transferase alpha 1/2/5 (GSTA1/2/5) were upregulated in male mice livers treated with ibuprofen compared to controls (Fig. 7A, Supplementary Table 1). To independently validate the results, the expression levels of GSTT2, GSTM1 and GSTA1/2/5 were analyzed by Western blotting. The expression of GSTM1 and GSTA1/2/5 was significantly upregulated in ibuprofen-treated male livers consistent with the proteomic data (Fig. 7B). In female ibuprofen-treated livers only the expression of GSTA1/2/5 was upregulated (Fig. 7C). Although not statistically significant, a trend towards increased GSTT2 (male) and GSTM1 (female) expression was observed in ibuprofen treated groups relative to control groups (Fig. 7B,C).

## Discussion

It is well acknowledged that high dose and long term use of NSAIDs are associated with adverse effects that include cardiac, gastrointestinal, renal and hepatotoxicity^32,33^. The investigation of male mice liver using mass spectrometry suggests that even moderate dosages of ibuprofen treatment affect various critical cellular pathways. Since liver is a major player in metabolism, this approach also provides mechanistic quantitative information on the molecular pathways that are affected by drug toxicity^34^. Various NSAIDs such as bromfenac, benoxaprofen and lumiracoxib were withdrawn from the market due to severe hepatotoxicity^35,36^. The incidence rate of drug-induced liver injury in outpatients in France was found to be fourteen per 100000 patients^37^. However, this is considered an underestimation as it will be unlikely to be detected in smaller clinical trials, and a more recent study in 2010 found that NSAIDs including ibuprofen (odds ratio of 2.24, 95% confidence interval (CI) = 1.99–2.52) increased the risk of hospitalization due to acute hepatitis (odds ratio 2.13, 95% CI = 2–2.18)^35^. The frequency of drug-related hepatic injuries, including from NSAIDs, was found to be different in Spain and France suggesting that several factors besides the NSAIDs, such as drug use patterns and/or genetic and environmental factors, may be important^36^.

Upadhyay *et al*., showed that ibuprofen induced proteasome dysfunction, mitochondrial-mediated apoptosis, and cell death in A549 and/or Cos-7 cells^38^. However, the cell death, proteasome inhibition and apoptosis were observed using 1 mM ibuprofen, which is significantly higher than the peak ibuprofen concentrations found in human blood (58.4 µg/mL or 283.09 µM) after high doses of ibuprofen^39^, and as such may not be physiologically relevant. The proteomic results suggested that the immunoproteasome subunit β5i was decreased in livers from ibuprofen treated male mice and Western blotting and proteasome assays suggest gender-specific differences with respect to proteasome and immunoproteasome activities. The proteasome is part of the ubiquitin proteasome system (UPS), which is critical for protein quality control and is the main intracellular degradation pathway that eliminates polyubiquitinated misfolded and oxidized proteins by a multi-complex 26 S proteasome^16,17,24,40^. The 26S mainly degrades polyubiquitinated proteins in an ATP dependent manner and contains caspase-like, trypsin-like and chymotrypsin-like proteolytically active sites in the β1 (PSMB6), β2 (PSMB7), and β5 (PSMB5) subunits of the 20S core^24,41^. On induction with interferon-γ (IFN-γ), the constitutive β1, β2 and β5 subunits of 20S proteasome are replaced by catalytically active inducible β1i (PSMB9), β2i (PSMB10) and β5i (PSMB8) subunits referred as the immunoproteasome^24,25^. The immunoproteasome plays an important role in degrading oxidized proteins, preventing protein aggregation, cytokine production, antigen presentation and T cell differentiation, and survival^24,42^.

The expression of the immunoproteasome β5i, which is important for the degradation of oxidized proteins under stressful conditions, was decreased in ibuprofen treated male livers relative to controls whereas it was increased in female mice livers in ibuprofen treated group. Despite the decrease in β5i expression in male livers and increased expression of β5i and β1i in female livers, the 26S β5i and β1i immunoproteasome activities remain unchanged in both ibuprofen treated male and female livers. Altered proteasome function is typically associated with increased levels of oxidized proteins and these possibilities were investigated in both male and female livers. The levels of oxidized proteins decreased in ibuprofen treated male livers compared to controls, while not significant, a trend towards the increase in levels of oxidized proteins (P = 0.086) was observed in ibuprofen-treated female livers relative to controls. A substantial decrease in β1 proteasome activity and β5i expression suggest altered proteasome functions in male livers. The elevated expression of 20S proteasome (increased PSMA6), which play an important role in the elimination of oxidized proteins, might be one of the mechanisms that account for reduced levels of oxidized proteins in male livers. However, in female livers increased β1, β1i and β5i expression, as well as a trend towards the increase in proteasome activities (β2, β1i, β5i) and β2i expression, suggest that ibuprofen activated a compensated stress response in female livers as a mechanism to adapt to various stressors. It has been previously documented that increased oxidative stress regulates proteasome and immunoproteasome functions^43^. Altogether these results suggest that ibuprofen causes proteasome dysfunction in both mice genders, male mice respond differently than female mice with respect to proteasome function.

### Proteins involved in antioxidant defense system

Mass spectrometry data showed that various antioxidant enzymes such as GSTT2, GSTM1 and GSTA1/2/5 that helps to eliminate toxic metabolites generated by oxidative stress were upregulated in response to ibuprofen treatment in male mice livers than controls. The expression levels of GSTM1 and GSTA1/2/5 (significant) was higher in ibuprofen-treated male livers. Similarly, in female ibuprofen-treated livers the expression of GSTA1/2/5 was upregulated significantly. GSTT2 (male) and GSTM1 (female) showed a trend toward higher levels relative to respective controls. Catalase activity, an important antioxidant activity, showed higher levels in both male and female livers. Increased levels of H_2_O_2_ was observed in both ibuprofen treated male and female livers relative to controls. During oxidative stress and other stress, conditions increased levels of H_2_O_2_ is associated with elevated production of antioxidant enzymes as a defense mechanism^31^ that explains the upregulation of anti-oxidant pathways in both male and female livers treated with ibuprofen. Also, the glutathione system plays regulatory role to maintain redox state of the cell^44^.

### Proteins involved in energy metabolism

Mass spectrometry showed that proteins associated with metabolic processes are the largest group of differentially expressed proteins that occur in response to ibuprofen treatment in male livers relative to controls. Western blotting showed that two of the three key enzymes involved in regulating glycolysis, pyruvate kinase L/R-type (PKLR) and phosphofructokinase 1 (PFK-1), were downregulated in male livers compared to controls. In contrast, PKLR was upregulated while no difference in the levels of PFK-1 was observed in ibuprofen treated female livers. PKLR is an enzyme responsible for catalyzing the conversion of phosphoenolpyruvate and adenosine triphosphate (ATP) to pyruvate and ATP in glycolysis^45^. PFK catalyzes the rate-limiting phosphorylation of fructose-6-phosphate to fructose-1,6-diphosphate. Downregulation or deficiency of pyruvate kinase leads to ATP depletion which in turn affects cell viability as well as accumulation of the glycolytic intermediates (2,3-diphosphoglycerate (2,3-DPG) that eventually leads to impaired glycolytic flux^46^. In isolated perfused rat livers mefenamic acid was reported to stimulate glycolysis, oxygen uptake, glycogenolysis and fructolysis^47^. PFK null mice were characterized by reduced lifespan, exercise intolerance and progressive cardiac hypertrophy^48^. Thus, the lower expression of PKLR and PFK-1 suggest that the ibuprofen affects metabolism and altered hepatic glucose metabolism in male mice. The upregulation of PKLR in female livers suggests accelerated glycolysis as an adaptive response to compensate for the depletion of ATP against oxidative stress.

Differential changes in PKLR in males (decreased) and females (increased) could have significant functional consequences in liver since glycolytic inhibition (such as by decreases in PKM2) has been suggested to promote the pentose phosphate pathway to generate higher levels of NADPH. NADPH is important in recycling oxidized glutathione and provides the reducing power needed for protein-based antioxidant systems^49^. ATP5F1 is part of the ATP synthase complex which generates ATP. ATP5F1 was reduced in male mice and increased in female mice compared to controls. In some cell types reduced ATP levels have been found to promote oxidative stress^50^. The electron transport chain is also a major site for superoxide production^51^. These results support several publications which suggest that oxidative stress is higher in male rats than female rats^52,53^.

A deficiency in cellular ATP has been suggested to cause several diseases^54^. In a mouse model of Leigh syndrome caused by a deficiency in a component of complex I, the levels of ATP were observed to dictate disease severity and neuronal death^55^. Intracellular ATP concentrations have also been shown to be important in intracellular protein degradation with the proteasomal activity being regulated by ATP levels^56^. Importantly, impaired bioenergetics affects the redox balance of a cell^57^. ROS can also induce bioenergetic changes in cells and tissues, such as in liver where ROS induced changes in glucose and lipid metabolism^58^.

The mass spectrometry data also suggest that other metabolic pathways are affected, and these include pathways previously reported and summarized by Porter *et al*.^59^, such as the pentose phosphate shunt and the electron transport chain. Evidence for NSAIDs altering other pathways such as cholesterol^60^, and vitamin^61^ metabolic pathways all support the proteomic data. Other pathways that were found to be affected by NSAIDs that were not previously reported include steroid, phospholipid, sulfur compound, and amino acid metabolic pathways.

### Proteins involved in fatty acid metabolism

#### Proteins involved in fatty acid biosynthesis

Glucose and fatty acids are metabolized in the liver and regulate whole-body energy homeostasis^7^. At high glucose levels, glycolysis is dominant and glycolytic intermediates and products are utilized to generate ATP as well as lipids and amino acids. During energy deprivation (low glucose levels), hepatocytes switch from glycolysis to fatty acid β oxidation for energy production^7^. Fatty acid oxidation depends upon the plasma concentration of free fatty acids and occurs in three subcellular organelles, the mitochondria and peroxisomes which carried out β-oxidation, whereas ώ-oxidation catalyzed by CYP4A1 takes place in endoplasmic reticulum^62^. FAS is an important enzyme of fatty acid biosynthesis which in the presence of NADPH catalyzes the condensation of acetyl-CoA and malonyl-CoA to generate palmitic acid^63^. In normal healthy tissues, FAS is expressed at very low levels. High expression of FAS is correlated with molecular changes in various types of cancer cells including breast cancer^64–66^. Ibuprofen treatment stimulated higher expression of FAS in both male and female livers suggesting altered fatty acid metabolism.

#### Proteins involved in fatty acid oxidation

In liver, fatty acids are metabolized by two major metabolic pathways; they either undergo β-oxidation in mitochondria and peroxisomes to produce ATP for energy requirements or esterified to triglycerides to generate either lipoproteins to export or lipid droplets for storage. The short, medium and long fatty acid chains are oxidized in mitochondria whereas very long chain fatty acids are degraded by peroxisomes^67^. Kasuya *et al*. reported that various NSAIDs (diflunisal, enoxacin, salicylic acid, aspirin and ofloxacin) and quinolone antimicrobial drugs have inhibitory effects on the medium chain acyl-CoA synthetase in mouse and bovine liver mitochondria^68^. LFABP regulates the lipid β-oxidation in mammalian liver and intestine and is correlated with various disease conditions including obesity, type 2 diabetes, insulin resistance, and fatty liver disease in humans^69,70^. Elevated expression of LFABP in ibuprofen treated male and female livers together with increased levels of H_2_O_2_ (a byproduct of fatty acid β-oxidation) is indicative of increased fatty acid metabolism. FAAH is a membrane-bound enzyme that plays an important role in breaking endogenous fatty acid amides such as the endogenous cannabimimetic agent anandamide (AEA) and palmitoylethanolamide (PEA) (an anti-inflammatory agent)^71^. NSAIDs such as aspirin, ibuprofen, and indomethacin were reported to have inhibitory action on FAAH with different potencies. Ibuprofen was shown to attenuate the metabolism of AEA by FAAH in rat brain^71^. In this study ibuprofen significantly enhanced FAAH expression in male livers while no significant change in female livers was observed. Hence, gender-specific differences in fatty acid metabolism were observed in ibuprofen treated mice.

The CYP4A1 and 4A2 gene expression have been reported to increase with NSAIDs^72–74^. CYP1-4 enzymes play an important role in the oxidative metabolism of fatty acids, steroids, polycyclic aromatic hydrocarbons (PAHs), various drugs such as acetaminophen, caffeine, clozapine, phenacetin, clomipramine and naproxen, and other carcinogens and environmental pollutants^73,74^. When treated with ibuprofen male livers showed no change in CYP1A2 and elevated levels of CYP4A1 while female livers showed significantly elevated expression of CYP1A2 and lower expression of CYP4A1. The opposite effects of ibuprofen on CYP4A1 in male and female mice support previous publications which suggest that sex related differences occur with respect to drug metabolism and adverse reactions. Females have been shown to give a 1.5- to 1.7-fold higher risk factor for having adverse drug reactions than men^75^. Although the underlying mechanism for these gender effects is not known, some of the differences have been attributed to the levels of sex hormones and their receptors^76^. However, some studies suggest that estrous variability is no greater in females than the intrinsic variability in males^77^. Some of the sex differences has also been linked to the expression of genes encoded by the X chromosome^78^. Other data also suggest that sex differences could occur at specific ages and in certain environments^79^. As such, we cannot assume that the sex related differences observed in this study is similar to what occurs at other ages. In these current studies, the sex-related differences observed may be partly due to sex-related differences in drug-metabolizing enzymes and drug transporters. The difference in the levels of CYP enzymes may also be related to the specific function of individual P450 enzymes and substrate specificity^73^. Moreover, oxidative stress and inflammatory factors can modulate CYPs transcription^80^. The increased oxidative stress in males could explain the lower expression of CYP4A1 in response to ibuprofen treatment. Although differential sex-related gene or protein expression of CYP4A1 has not been previously reported, many CYPs including CYP7A1 and CYP1B1 have been reported to show sex-related gene expression patterns^81,82^.

CROT is a carnitine acyltransferase that plays crucial roles in lipid metabolism and fatty acid β-oxidation^83,84^. Although localized to peroxisomes, CROT is essential for transport of medium and long length acyl-CoA chains from peroxisomes to the cytosol and mitochondria for further degradation^83^. The high expression of CROT in male (statistically significant) and female (trend to increase) livers suggest that it is possible that ibuprofen treatment induces peroxisomal oxidation of medium and long chain fatty acids metabolism as a compensatory mechanism due to an overload of β-oxidation of fatty acids in mitochondria.

### Other pathways proteins

17β-HSD2 is a microsomal enzyme that catalyzes the oxidation of sex steroids testosterone and estradiol (E2) to less active ketones androstenedione and estrone (E1)^85,86^. It is reported that the inhibition of 17β-HSD2 in a *in vivo* monkey model and osteoporosis patients is beneficial to increase in E2 and testosterone levels that facilitate reduction in bone resorption and maintenance of bone formation^87^. The gender-specific effect of ibuprofen in our study suggests that ibuprofen has more pronounced effect on female livers as higher 17β-HSD2 expression indicate more accumulation of sex hormone in females that will eventually lead to perturbed energy metabolism pathways. The age and sexual maturity of the mice used may also be important in the levels of the enzymes involved in sex hormone biosynthesis and may be partly responsible for some of the observed gender specific differences.

### Limitations of the study

Our study observed gender-specific ibuprofen-mediated effects on mice liver. However, since the proteomic profiling was only done on male mice, the liver proteins and pathways investigated by Western blotting and biochemical assays were limited only to proteins which were identified as differentially regulated in liver of male mice. It is likely that female-specific changes in protein expression also occur in ibuprofen treated livers and these changes were not investigated. The effects observed may also be age specific and could be significantly different in mice of different ages.

## Conclusion

Mass spectrometry showed that moderate levels of ibuprofen given to healthy mice for seven days resulted in over 300 proteins that were significantly altered when compared to control groups. Western blotting and biological assays showed that livers from male and female mice showed differences in the UPS, glycolysis and fatty acid metabolism. It is likely that many of the other pathways that were affected by ibuprofen also show gender differences, but these pathways were not investigated in this study. The data also suggests that ibuprofen affects previously unknown pathways such as amino acid, steroid and vitamin metabolism. Overall, our data indicate that moderate doses of ibuprofen can affect liver more significantly than previously reported and include proteasome dysfunction, increased levels of H_2_O_2_, impaired glycolytic pathways and altered fatty acid synthesis and oxidation. Although we expected similar differences between males and females with respect to ibuprofen treatment, complex gender-specific ibuprofen-mediated effects were observed. These gender-specific effects may be important for the side effects of ibuprofen (such as increased risk of stroke) and more research is needed to investigate this possibility. The amounts of ibuprofen taken by males and females may need to be altered in the future. Although the published experimental data to support that males and females show important physiological differences that affect the pathophysiology of many common diseases is increasing at a steady pace, very little gender-specific health care is utilized worldwide. Ultimately, gender should be considered in drug use, as the toxicity of some drugs is significantly different between women and men.

## Supplementary information

Supplementary Information.

Supplemental Table 1.

Supplemental Table 2.

## Data Availability

Raw data, and Scaffold results are available from the MassIVE proteomics repository (MSV000083987) and Proteome Exchange PXD014279. All Western Blot raw data generated or analyzed during this study are included in this published article (Supplementary Figure 4). The datasets generated during the current study are available from the corresponding author on reasonable request.
