# Supplementary material for: Gender-specific changes in energy metabolism and protein degradation as major pathways affected in livers of mice treated with ibuprofen
Source: Sci Rep. 2020 Feb 25;10:3386. doi: 10.1038/s41598-020-60053-y (PMC7042271; doi:10.1038/s41598-020-60053-y)
Supplement: Supplementary file 1 — Supplementary Information. [file 41598_2020_60053_MOESM1_ESM.pdf]

Supplementary Information.

Shuchita Tiwari, Manish Mishra, Michelle Salemi, Brett S. Phinney, Joanne L. Newens and Aldrin V. Gomes. Gender-specific changes in energy metabolism and protein degradation as major pathways affected in livers of mice treated with ibuprofen. Scientific Reports

Proteasome Assays

Proteasome and immunoproteasome assays to verify inhibitor and substrate effectiveness were carried out using purified 26S proteasomes and 20S immunoproteasomes (obtained from Boston Biochem Inc., Cambridge MA) (Supplementary Figure 4).

TMT data analysis (Quantitative data analysis)-

For data analysis channels were corrected by the matrix

[0.000,0.000,0.000,0.000,0.926,0.000,0.0720,0.000,0.00200];
[0.000,0.000,0.000,0.00400,0.921,0.000,0.0730,0.000,0.00200];
[0.000,0.000,0.00500,0.000,0.932,0.000,0.0630,0.000,0.000];
[0.000,0.000,0.00700,0.000,0.934,0.000,0.0590,0.000,0.000];
[0.000,0.000,0.0140,0.000,0.935,0.000,0.0510,0.000,0.000];
[0.000,0.000,0.0250,0.000,0.925,0.000,0.0500,0.000,0.000];
[0.000,0.000,0.0230,0.000,0.934,0.000,0.0430,0.000,0.000];
[0.000,0.000,0.0270,0.000,0.934,0.000,0.0390,0.000,0.000];
[0.00400,0.000,0.0290,0.000,0.934,0.000,0.0330,0.000,0.000];
[0.000,0.000,0.0340,0.000,0.933,0.0330,0.000,0.000,0.000] in all samples according to the algorithm described in i-Tracker [1].

Reference

1 Shadforth, I. P., Dunkley, T. P., Lilley, K. S. & Bessant, C. i-Tracker: for quantitative proteomics using iTRAQ. *BMC Genomics* **6**, 145, doi:10.1186/1471-2164-6-145 (2005).

Supplementary Figures:

Supplementary Figure 1. Proteasome and Immunoproteasome assay controls. (A) 26S proteasome (5nM) in the presence of the same substrates and inhibitors used for liver proteasome assays. (B) Immunoproteasome in the presence of the same substrates and inhibitors used for liver immunoproteasome assays. (C) Sample results from control (CTRL) male and female livers in the

presence of β5 substrate and inhibitor. 26S, human 26S proteasome, IP, murine immunoproteasome, BKG, background fluorescence not due to proteolytic activity. INB, inhibitor.

Supplementary Figure 2. Validation of expression levels of mitochondrial proteins in male and female mouse liver. Changes in level of ATP5F1 (Mitochondrial ATP synthase 5F1) Prohibitin-2, and ETFDH (Electron transfer flavoprotein dehydrogenase) obtained by mass spectrometry (A). Western blots and corresponding semi-quantitative bar graphs for proteins, ATP5F1, the inner mitochondrial membrane proteins Prohibitin-2, and ETFDH and total protein (used as a loading control). (B) Male liver and (C) Female liver. Value are mean ± SE; n = 4-6 per group. *$p < 0.05$.

Supplementary Figure 3. Schematic diagram showing expression changes in enzymes involved in fatty acid β-oxidation in mitochondria and peroxisomes.

Abbreviations: CROT (carnitine O-octanoyltransferase); ETFDH (electron transfer flavoprotein dehydrogenase); ATP (Adenosine triphosphate)

Supplementary Figure 4. Uncropped western blots of the blots presented in the manuscript. Blots were cut into strips at 37 or 50 kDa and then the Western blotting procedure carried out.

Supplementary Tables:

Supplementary Table 1. Table showing all proteins quantified in livers from ibuprofen and vehicle-treated mice.

Supplementary Table 2. Table showing proteins that show a ≥ [1.2] fold change in protein expression in livers from ibuprofen and vehicle-treated mice.

Supplementary Table 3. Table showing the number of mice used per group for the different experiments.

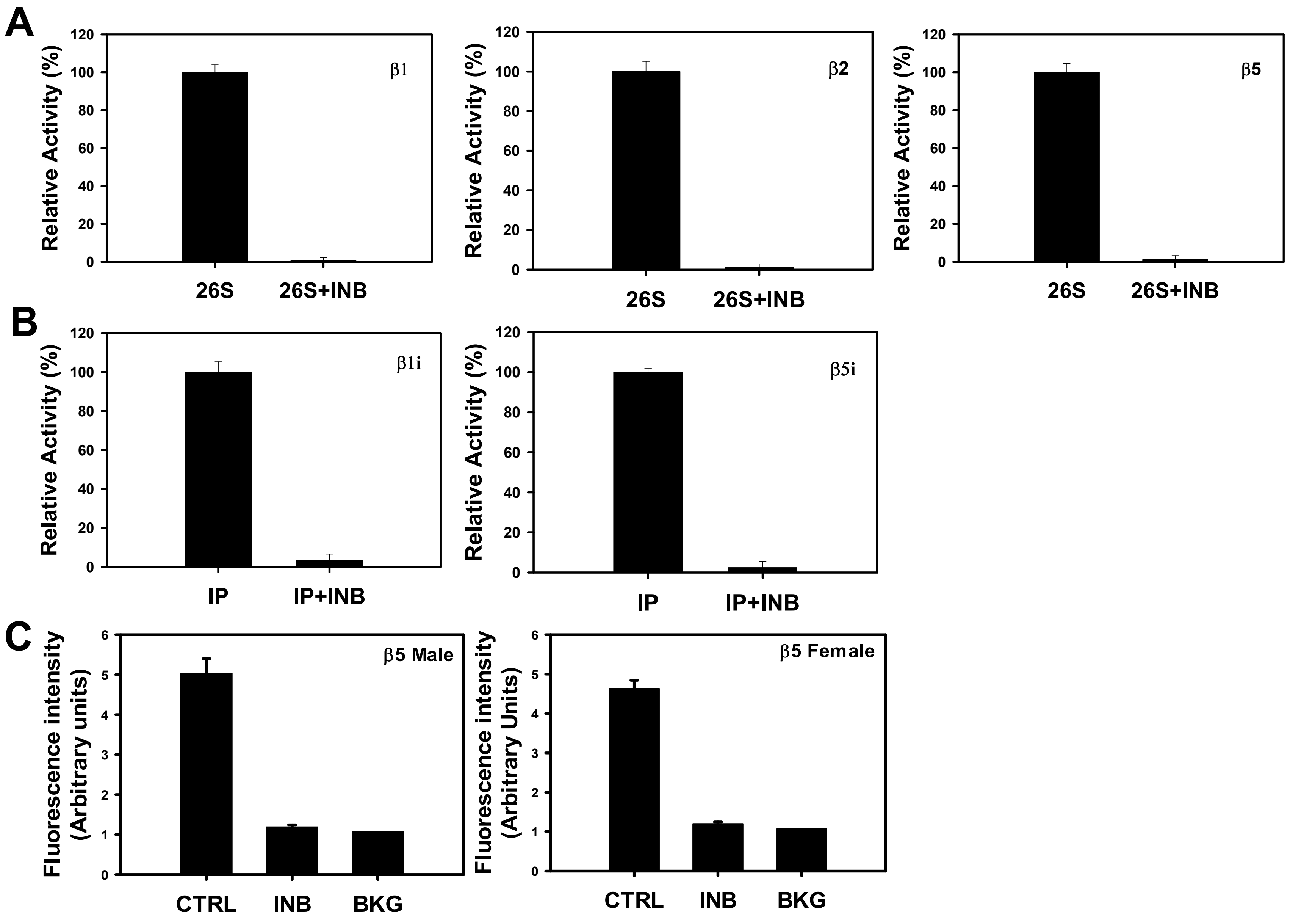

Suppl Figure 1

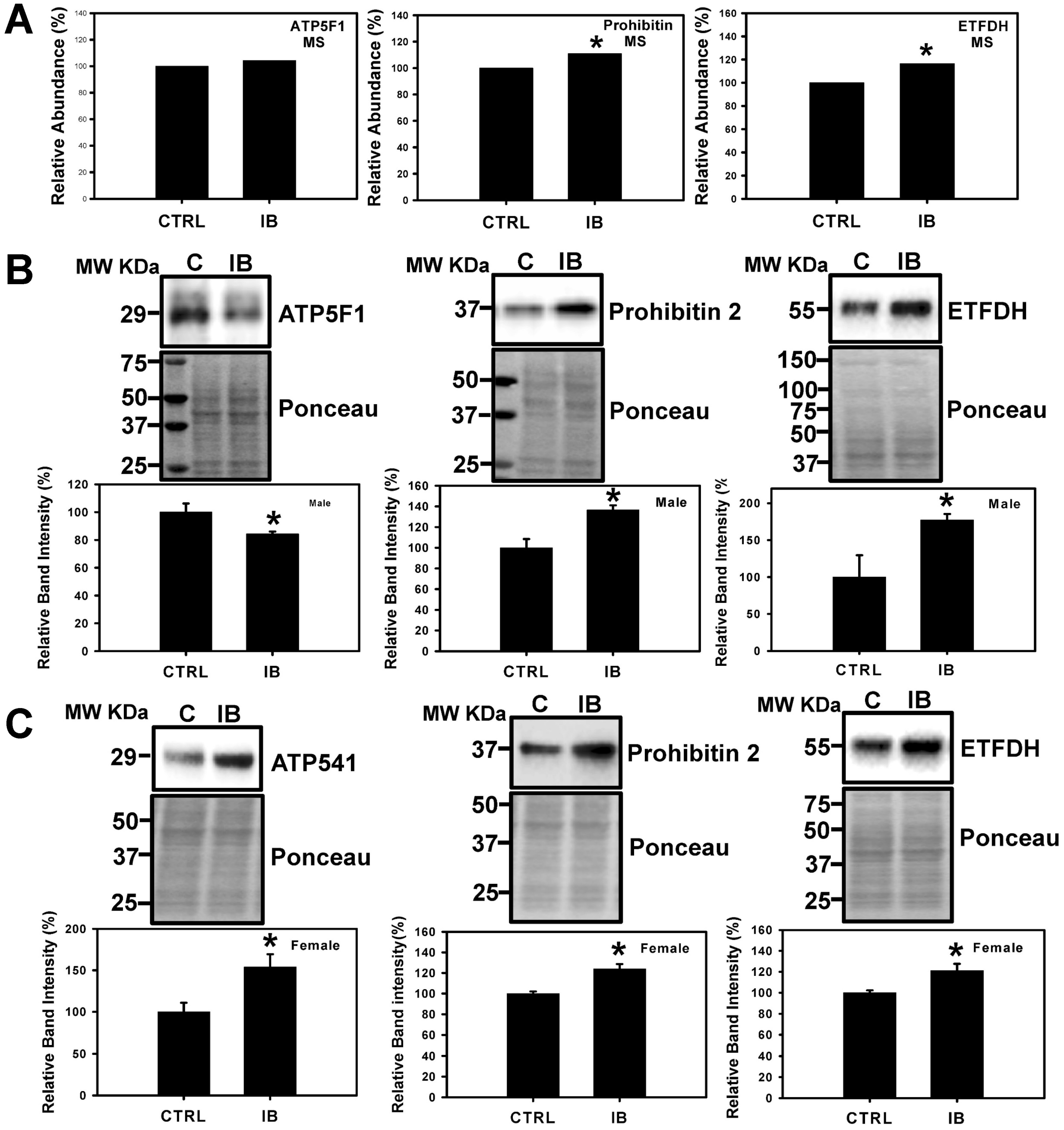

Suppl Figure 2

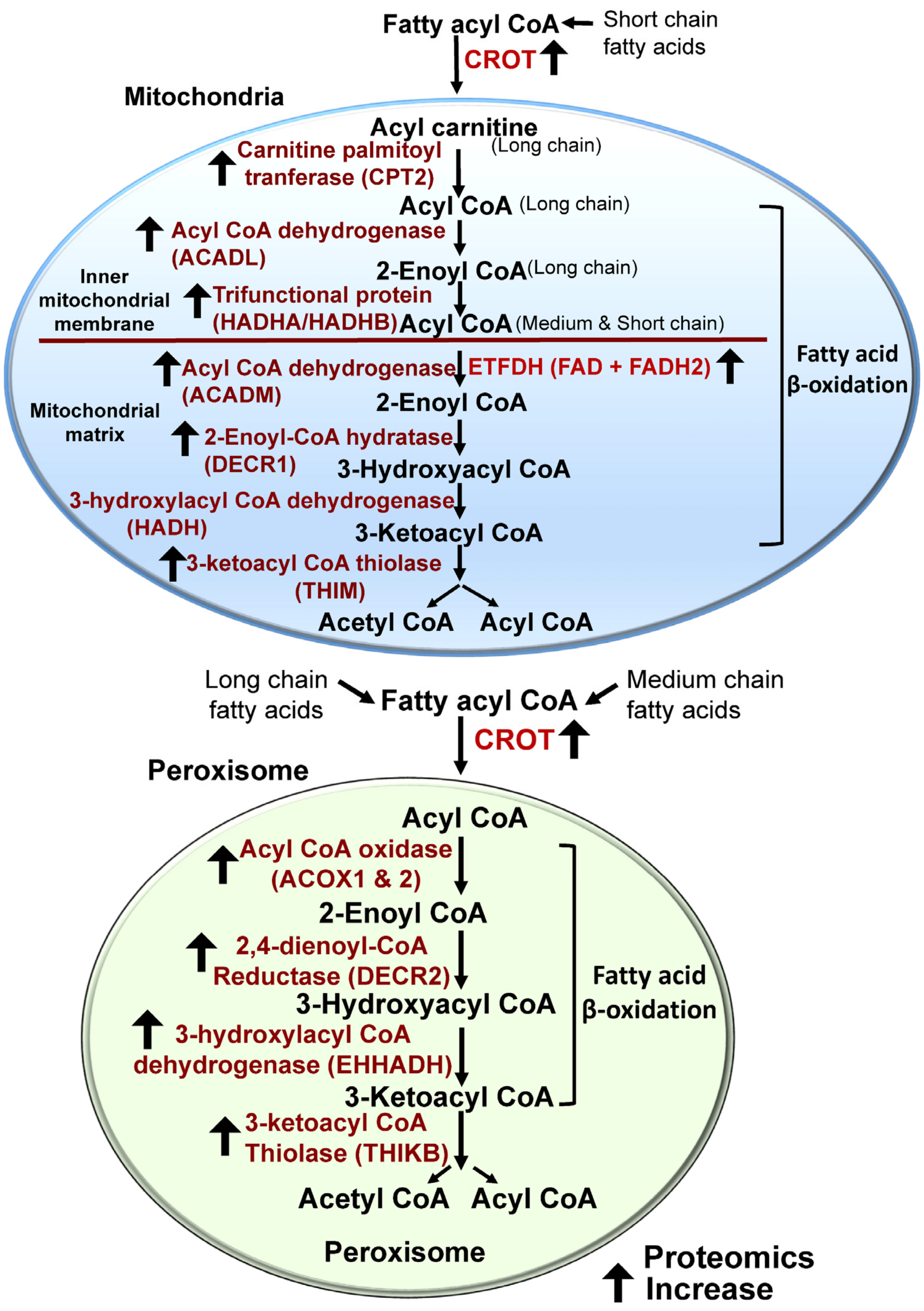

Suppl Figure 3

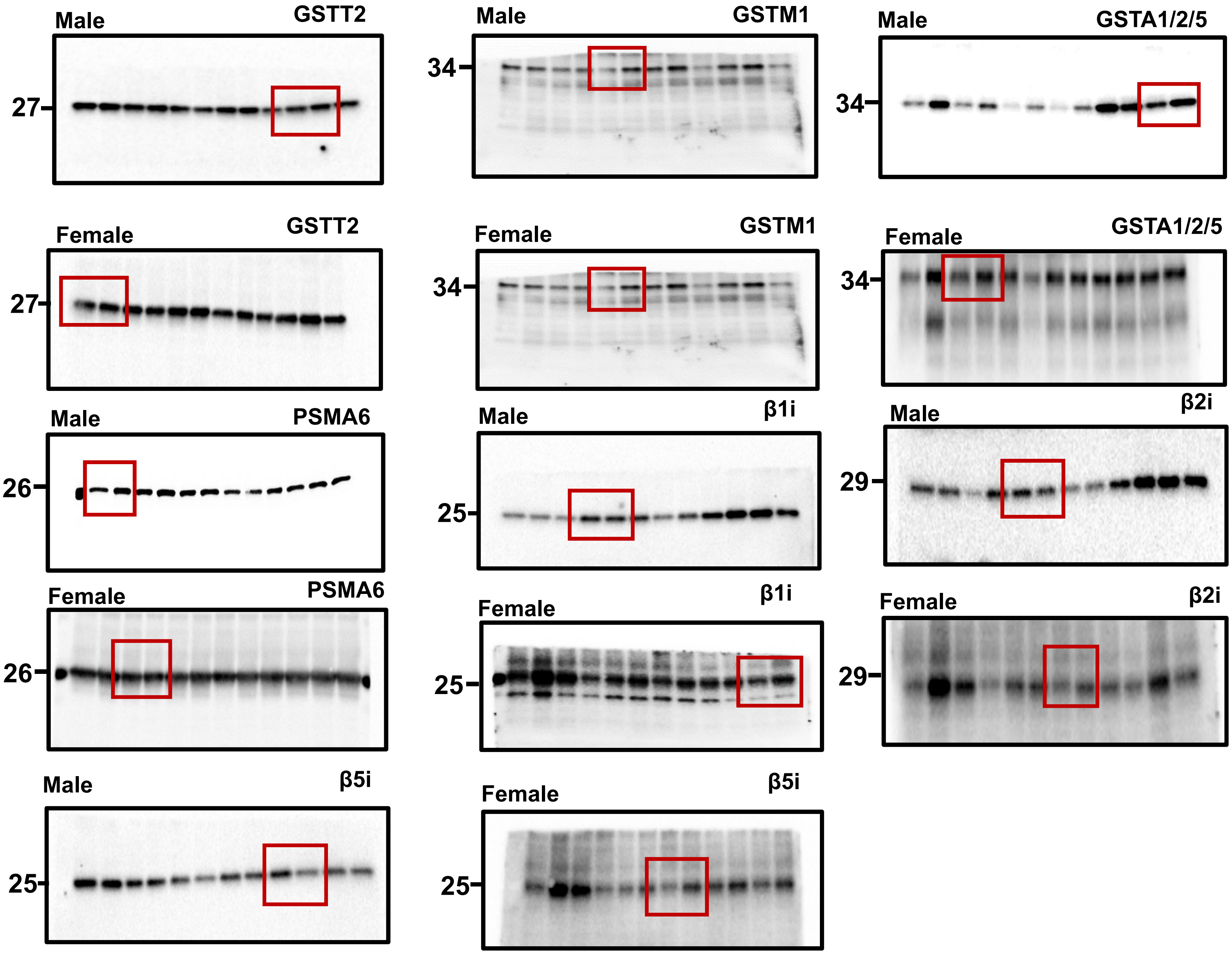

Suppl Figure 4

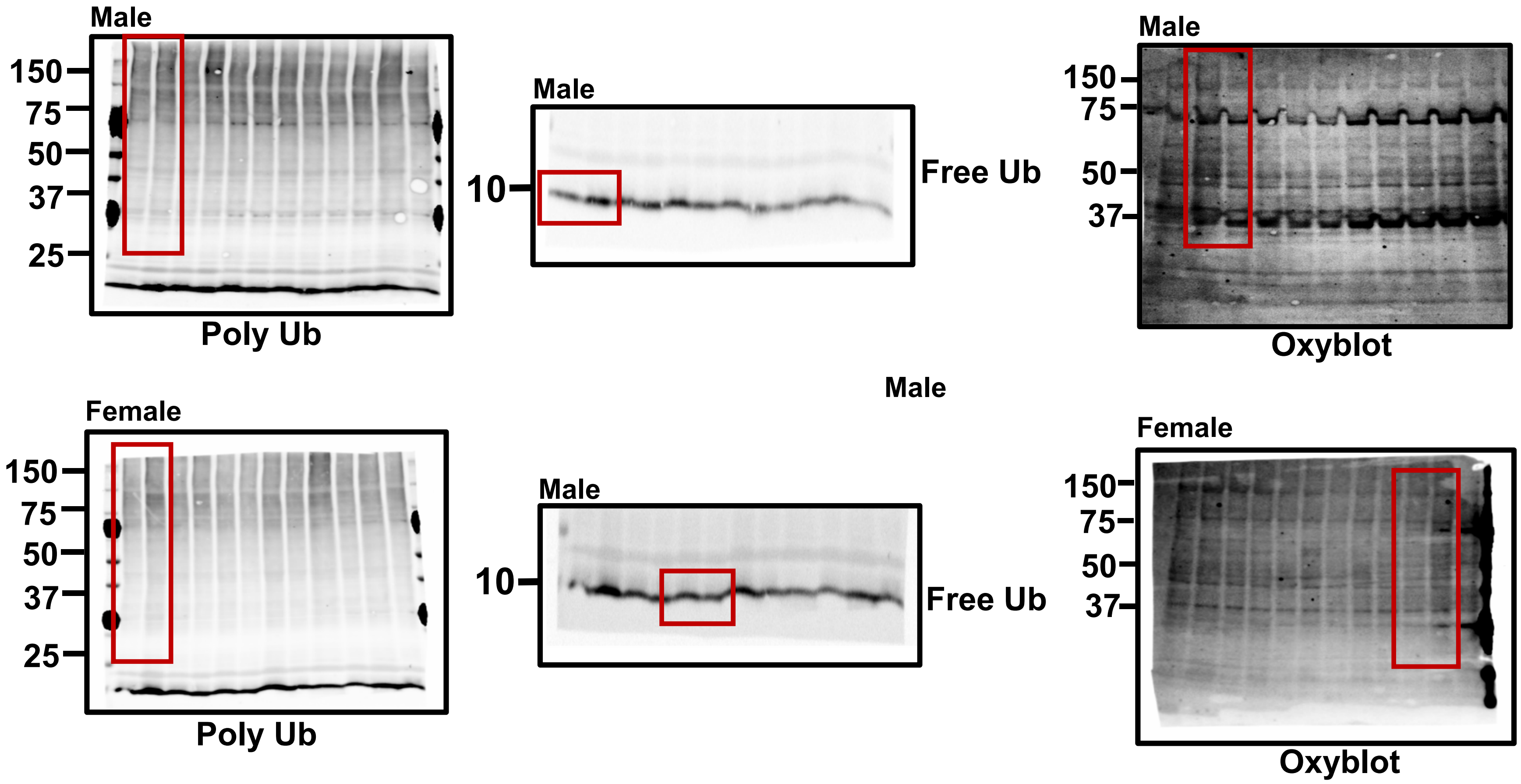

Suppl Figure 4

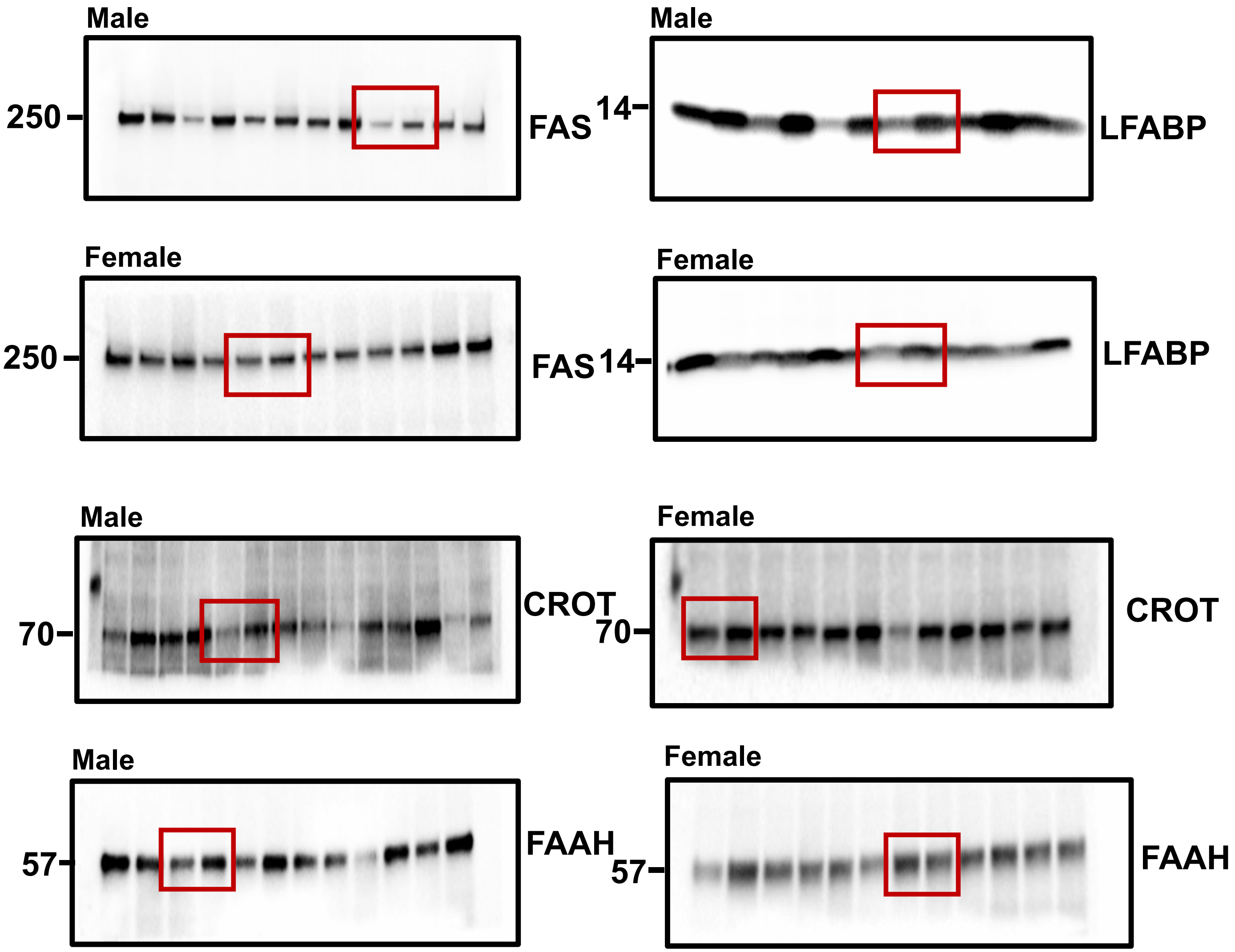

Suppl Figure 4

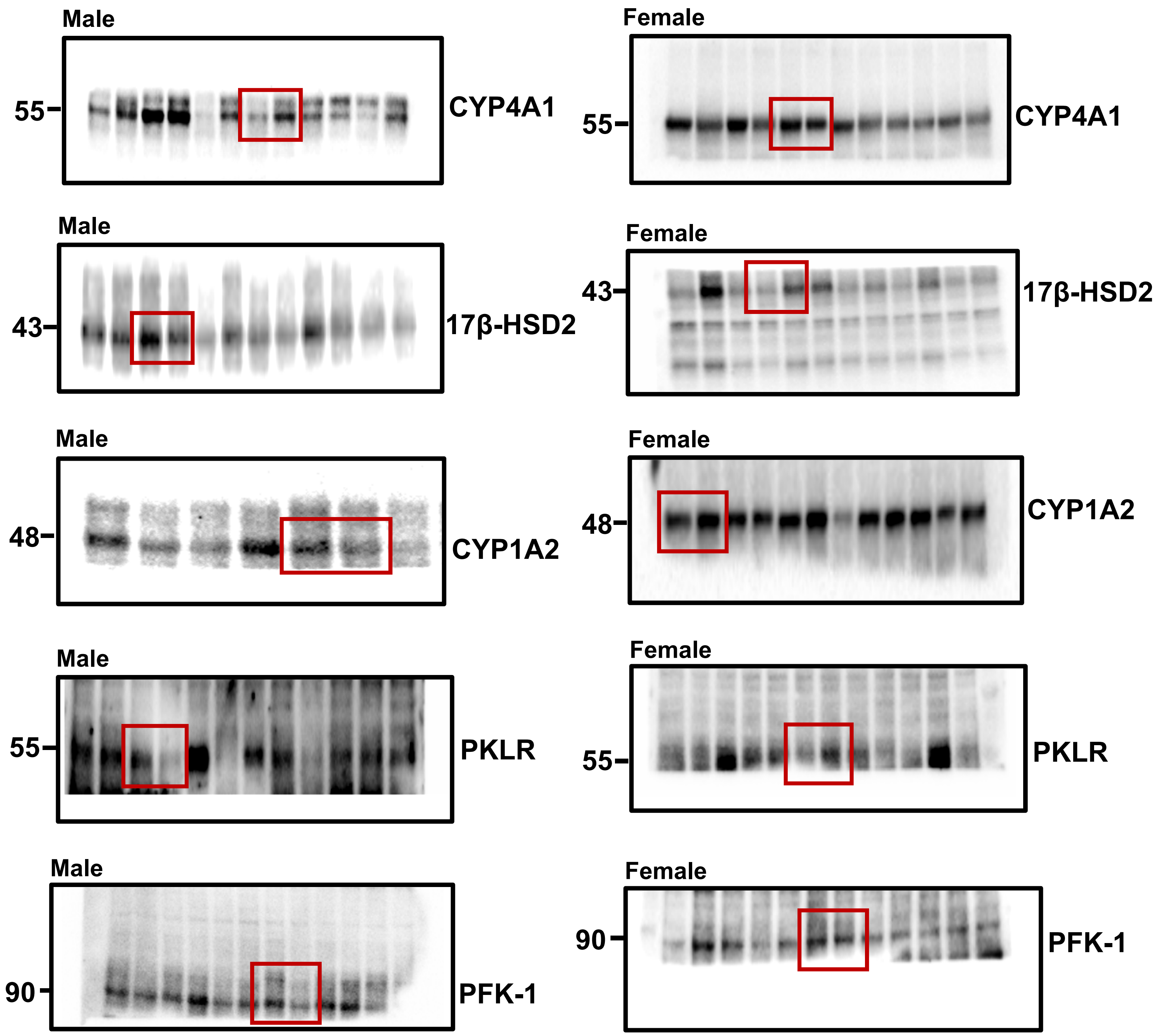

Suppl Figure 4

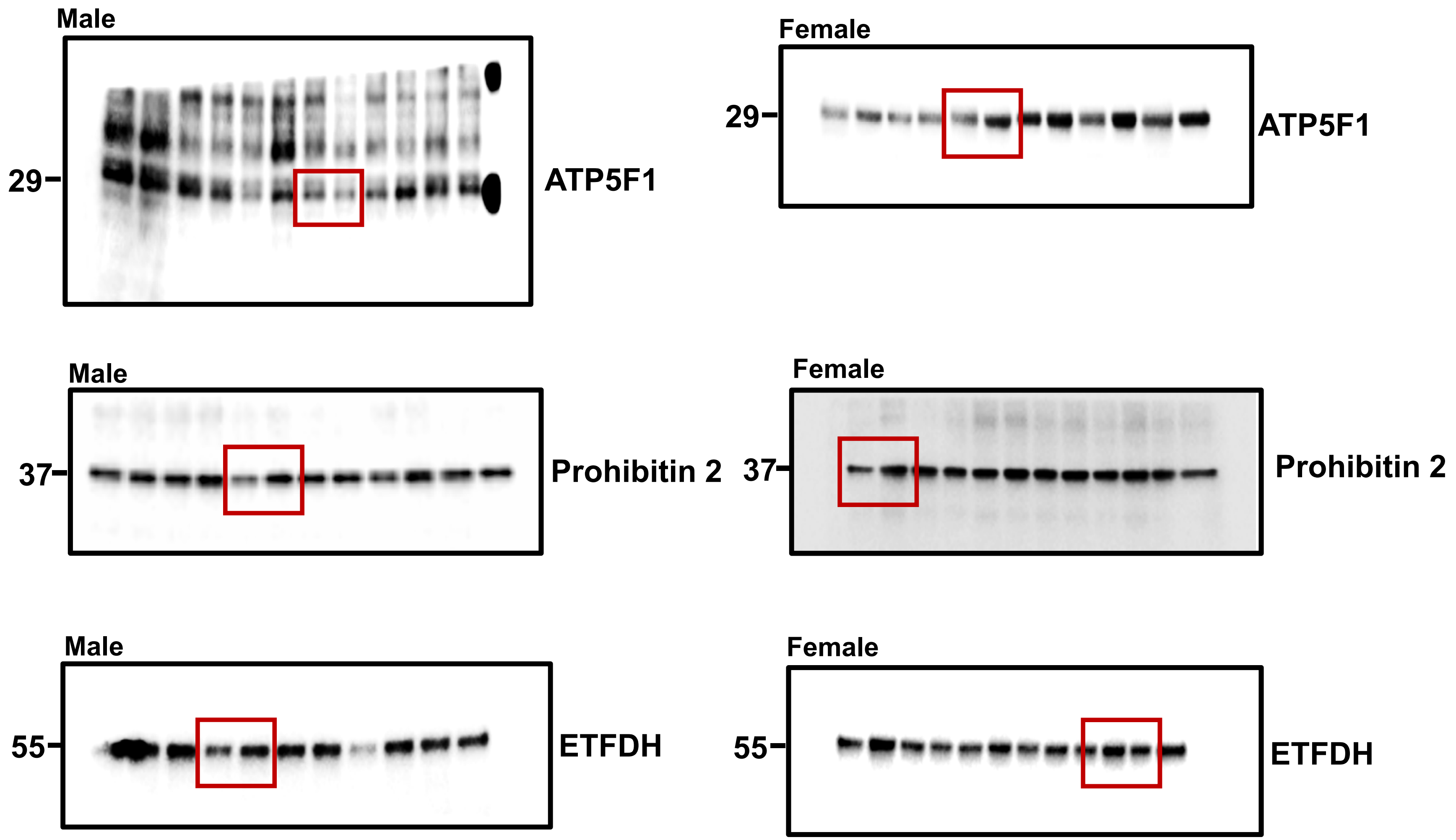

Suppl Figure 4

Supplementary Table 3: Table showing the number of mice used per group for the different experiments.

	Male (n)		Female (n)	
**Methods**	**Control**	**IB**	**Control**	**IB**
**Proteomics**	5	5	5	5
**Western blotting**	4-6	4-6	4-6	4-6
**Proteasome assay**	6	6	6	6
**Immunoproteasome assay**	6	6	6	6
**Catalase activity assay**	5	5	5	5
**Hydrogen peroxide assay**	5	5	5	5
